# “Prevalence of orthorexia nervosa in a sample of patients attending Sligo/Leitrim mental health services with a diagnosis of eating disorder”

**DOI:** 10.1192/bjo.2021.674

**Published:** 2021-06-18

**Authors:** Ignazio Graffeo, Mary Harron, Edmond O'Mahony

**Affiliations:** Sligo/Leitrim Mental Health Services – HSE

## Abstract

**Aims:**

The main aim of this study is to investigate its presence in a sample of patients already diagnosed with a canonical eating disorder and also to understand eventual overlaps with other clinical disorders in order to optimize treatment and follow-up. The ORTO-15 questionnaire, developed by an Italian team of researchers in 2005, was used to achieve the above aims: it is a tool comprehensive of 15 questions that assesses eating habits perceived as healthy. Really interesting and fascinating is to comprehend if people with a diagnosis of eating disorder present orthorectic behaviour and how this emerging reality fits in the Irish society with its peculiarities and uniqueness.

**Method:**

Every patient was asked to complete a demographic grid (elaborated by the researchers, which includes information regarding: age, gender, race, weight, height, hours of weekly exercise, years of education, employment situation, medical illnesses, smoking habits, type of diet, average weekly alcohol intake) and the Orto-15 questionnaire

**Result:**

The Point Prevalence obtained is 17.9%.

**Conclusion:**

The results obtained from this study give a clear indication of the profile of the orthorexic patient, considered that the sample was obtained from a population of people with a diagnosis of Eating Disorder:

- Caucasian woman in her 30s
- Exercising 5 hours per week
- Secondary education
- Unemployed
- Non-smoker
- Diagnosis of Anorexia Nervosa
- No other comorbid psychiatric illnesses
- Standard pattern of eating
- Minimal or absent alcohol consumption
- Normal range BMI

According to previous Italian studies (Ramacciotti et al. 2011), the expected rates of Orthorexia Nervosa in the general population are between 6.9% and 57.6%, with a peak of 81.8% in specific populations, fact that places our examined sample in the lower side of the prevalence previously considered. It is very difficult to comprehend and explain the reasons behind this fact and probably this is due to an overshadowing of symptoms with the major eating disorders. It is also significant the absence of correlation found between OCD and ON and also the fact that ON is more linked to Bulimia Nervosa in our sample rather then Anorexia Nervosa.

